# Virtual Simduction Programme Improves Junior Doctor Confidence and Knowledge for Psychiatry Rotation

**DOI:** 10.1192/bjo.2022.122

**Published:** 2022-06-20

**Authors:** Daniel Di Francesco, Robert Vaughn

**Affiliations:** Sussex Partnership NHS Foundation Trust, Eastbourne, United Kingdom

## Abstract

**Aims:**

To design a virtual simulation training session for junior doctors starting their psychiatry rotation, to be delivered virtually at induction. To measure how this changes doctors' confidence and knowledge about the rotation.

**Methods:**

A small committee of experienced psychiatric trainees decided on a set of 5 common on call scenarios. Focus was given to clinical scenarios that involve the use of good communication skills with patients and with other clinical staff encountered on call such as Nurses and HCAs.

The 5 stations focused on:

Using section 5(2), risk assessment

rapid tranquilisation

neuroleptic malignant syndrome

alcohol detoxification

managing self harm and ligatures.

Each scenario utilised real world documentation as tools for the candidate to utilise (drug charts, NEWS charts etc) to increase fidelity. Detailed actor briefs were drawn up with instructions for the facilitators.

During delivery of the sessions, a ‘safe learning space’ was set before individual learners took on the scenarios. ‘Time outs’ were utilised to allow the candidate to think through the scenario with the facilitator.

After each scenario, the facilitator then used crib sheets to lead ‘mini tutorials’ for 10 minutes around each scenario to flesh out the theoretical and practical learning points. The simulation-trained actors gave feedback on communication skills. Candidates were provided with feedback forms at the conclusion.

**Results:**

Feedback from the sessions was overwhelmingly positive. Every single candidate (n = 30) either agreed or strongly agreed that the session met the learning outcomes of increasing confidence, creating a realistic setting, being a fun and enjoyable introduction to psychiatric services.

Blank space feedback was also excellent, with many doctors asking for further expansion of the development of the session into a rolling program, and expansion of the scenarios to include more complex clinical situations that involve other members of the MDT.

**Conclusion:**

The virtual simduction programme is an effective way to improve confidence and knowledge of common scenarios faced for junior doctors new to a psychiatry rotation. Further development will involve a transition to a face-to-face programme and integration of the wider MDT, including nurses, support workers and pharmacists.

